# Clinical significance of glutamate metabotropic receptors in renal cell carcinoma risk and survival

**DOI:** 10.1002/cam4.1901

**Published:** 2018-11-28

**Authors:** Chao‐Yuan Huang, Yu‐Mei Hsueh, Lih‐Chyang Chen, Wei‐Chung Cheng, Chia‐Cheng Yu, Wei‐Jen Chen, Te‐Ling Lu, Kuo‐Jin Lan, Cheng‐Hsueh Lee, Shu‐Pin Huang, Bo‐Ying Bao

**Affiliations:** ^1^ Department of Urology, College of Medicine National Taiwan University Hospital, National Taiwan University Taipei Taiwan; ^2^ Department of Urology National Taiwan University Hospital Hsin‐Chu Branch Hsinchu Taiwan; ^3^ Department of Family Medicine Shuang Ho Hospital, Taipei Medical University New Taipei City Taiwan; ^4^ Department of Public Health, School of Medicine, College of Medicine Taipei Medical University Taipei Taiwan; ^5^ Department of Medicine Mackay Medical College New Taipei City Taiwan; ^6^ Graduate Institute of Biomedical Sciences China Medical University Taichung Taiwan; ^7^ Research Center for Tumor Medical Science China Medical University Taichung Taiwan; ^8^ Drug Development Center China Medical University Taichung Taiwa; ^9^ Division of Urology, Department of Surgery Kaohsiung Veterans General Hospital Kaohsiung Taiwan; ^10^ Department of Urology, School of Medicine National Yang‐Ming University Taipei Taiwan; ^11^ Department of Pharmacy Tajen University Pingtung Taiwan; ^12^ School of Public Health, College of Public Health Taipei Medical University Taipei Taiwan; ^13^ Department of Pharmacy China Medical University Taichung Taiwan; ^14^ Department of Urology Kaohsiung Medical University Hospital Kaohsiung Taiwan; ^15^ Department of Urology, Faculty of Medicine, College of Medicine Kaohsiung Medical University Kaohsiung Taiwan; ^16^ Graduate Institute of Medicine, College of Medicine Kaohsiung Medical University Kaohsiung Taiwan; ^17^ Institute of Biomedical Sciences National Sun Yat‐sen University Kaohsiung Taiwan; ^18^ Sex Hormone Research Center China Medical University Hospital Taichung Taiwan; ^19^ Department of Nursing Asia University Taichung Taiwan

**Keywords:** glutamate metabotropic receptors, prognosis, renal cell carcinoma, single‐nucleotide polymorphisms, survival

## Abstract

Accumulating evidence suggests the roles of glutamate metabotropic receptors (GRMs) in cancer, in addition to synaptic signalling. The present study assessed the associations of genetic variants in eight *GRM* genes with regard to risk and overall survival (OS) in 780 renal cell carcinoma (RCC) patients and controls. After adjustment for known risk factors, *GRM5* rs7102764 T was associated with an increased risk of RCC (*P* = 0.006). Additional analysis has provided evidence that rs7102764 T was correlated with a higher expression of *GRM5*, which is consistently found to be upregulated in tumours, compared to normal tissues. Furthermore, the *GRM3* rs701332 C, *GRM4* rs2499707 T, and *GRM4* rs4713742 T alleles were significantly associated with a poorer OS (*P* ≤ 0.030). The three loci were also observed to have strong cumulative effects on OS. Additional analysis has revealed a significant genotype‐expression correlation of rs2499707 T with increased *GRM4* expression, which in turn leads to poorer OS in patients with RCC. GRMs might be involved in RCC development and progression, and genetic variants in *GRM*s might be promising biomarkers.

## 1 INTRODUCTION

Renal cell carcinoma (RCC) is the most common type of kidney cancer, and is one of the most common cancers in the United States, with an estimate of 65 340 new cases and 14 970 deaths occurring in 2018.^1^ In Taiwan, kidney cancer was ranked as the 15th and 16th leading cause of cancer mortality in males and females, respectively, and accounted for 1.27% of all cancers in 2015. Multiple environmental risk factors, such as cigarette smoking and arsenic exposure, and genetic variations have been reported to be involved in the aetiology of RCC.^2^, ^3^ It is important to explore RCC‐related risk factors and its underlying mechanisms in order to improve therapeutic treatments.

Glutamate is an excitatory neurotransmitter in processes such as memory and learning,^4^ but recent studies have also implicated glutamate signalling in the development and progression of various cancers.^5^ Glutamate metabotropic receptors (GRMs) are G‐protein‐coupled receptors, which are activated by glutamate and stimulate secondary messengers such as phospholipase C/protein kinase C/calcium, phosphatidylinositol 3‐kinase/Akt/mammalian target of rapamycin, and mitogen‐activated protein kinase pathways, to generate the glutamate signalling cascades.^6^ The GRM family comprises eight members and is classified into three groups according to the difference in sequence similarity and downstream second messenger pathways.^7^ Group I (GRM1 and 5) initiates signalling via the phospholipase C/protein kinase C/calcium pathway, whereas group II (GRM2 and 3) and group III (GRM4, 6, 7 and 8) couple negatively with adenylyl cyclase to suppress the production of cyclic AMP and inhibit protein kinase A.

In addition to the central nervous system, a functional glutamatergic system has been reported in non‐neuronal peripheral cells.^8^ Furthermore, studies have suggested that glutamate signalling is dysregulated and may play roles in human malignancies.^9^ Here, we explored the associations of gene expression and genetic variants of *GRM*s with prognosis in patients with RCC.

## 2 MATERIALS AND METHODS

### 2.1 Patient population and clinical data collection

The study population consisting of 780 participants was recruited from three Taipei city hospitals: National Taiwan University Hospital, Taipei Medical University Hospital, and Taipei Municipal Wan Fang Hospital, as described previously.^2^, ^10^ A total of 390 patients with pathologically confirmed RCC were matched for age (±1 year) and gender with 390 cancer‐free controls. The demographic data were collected through in‐person interviews using a structured questionnaire, and the clinical and follow‐up information was obtained from medical records. Recurrence‐free survival (RFS) was defined as the time from surgery to the first date of recurrence. Overall survival (OS) was defined as the time from surgery to death due to any cause. This study was performed in accordance with the approval protocols by The Research Ethics Committee of National Taiwan University Hospital, and written informed consent was obtained from all participants before the questionnaire interview and specimen collection.

### 2.2 Single‐nucleotide polymorphism (SNP) selection and genotyping

The candidate SNPs were identified across eight *GRM* genes (*GRM1‐8*), including 5 kb upstream and 1 kb downstream of each gene, using SNPinfo.^11^ TagSNPs were selected based on a minor allele frequency (MAF) of >0.05 in the HapMap CHB (Han Chinese in Beijing) population, a pairwise linkage disequilibrium squared correlation coefficient (*r* ^2^) of >0.8, and whether they were potentially functional; a maximum of five tagSNPs per gene was selected. A total of 35 tagSNPs were selected for genotyping. Genomic DNA was extracted from peripheral blood samples using the QIAamp DNA Blood Mini Kit (Qiagen, Valencia, CA, USA). Genotyping was carried out as described previously,^12^ using Agena Bioscience iPLEX matrix‐assisted laser desorption/ionization time‐of‐flight mass‐spectrometry technology at the National Center for Genome Medicine, Taiwan. Any SNP that failed the assay design (N = 7), deviated from Hardy‐Weinberg equilibrium (*P* < 0.01, N = 2), or had an MAF of <0.01 (N = 1) was excluded. Finally, a total of 25 SNPs were included for further analysis, and the average genotype call rate was 98.8%.

### 2.3 Bioinformatics analysis

We used HaploReg v4.1 (https://pubs.broadinstitute.org/mammals/haploreg/haploreg.php) to annotate the regulatory features of the region adjoining the risk SNPs.^13^ The association of selected SNP‐gene expression quantitative trait loci (eQTL) was evaluated using Genotype‐Tissue Expression (GTEx).^14^ The clinical significance of the expression of *GRM*s on RCC was analysed using The Cancer Genome Atlas Kidney Renal Clear Cell Carcinoma (TCGA‐KIRC) data^15^ and DriverDB.^16^, ^17^

### 2.4 Statistical analysis

Chi‐square or Mann‐Whitney *U* test was used to compare the categorical or continuous variables, respectively, between the RCC cases and healthy controls. Univariate and multivariate logistic regression was used to estimate the crude and adjusted odds ratios (ORs) and 95% confidence intervals (CIs) of the SNP genotypes and RCC risk. Multivariate logistic regression models for each SNP were adjusted for age, gender, alcohol consumption, and histories of hypertension and diabetes. Kaplan‐Meier analysis with the log‐rank test was used to assess the associations of SNPs or gene expression with OS. Multivariable Cox regression, after adjustment for age and gender, was performed to estimate the adjusted hazard ratios (HRs) and their 95% CIs for the association of SNP with OS. Spearman's rank correlation tests were used to determine the association between the expression of *GRM*s and clinical characteristics of RCC. All statistical analyses were performed using Statistical Package for the Social Sciences (SPSS) software version 19.0.0 (IBM, Armonk, NY, USA), and a two‐sided *P* value of <0.05 was considered nominally significant. False discovery rate (*q* value) was calculated using the R‐package to adjust for multiple testing.^18^ As previously suggested, all SNPs with *q* < 0.20 were reported to account for multiple testing while balancing the discovery nature of our study.^19^

## 3 RESULTS

The clinical characteristics of 390 RCC patients and 390 age‐ and gender‐matched healthy controls are shown in Table 1. Age, gender, body mass index (BMI), and cigarette smoking status were comparable between RCC patients and control subjects. However, significant differences between the cases and controls were noted in case of alcohol consumption and histories of hypertension or diabetes (*P* < 0.001). Most RCC cases had stage I‐II and grade I‐II of the disease, and the median follow‐up time was 19.6 months.

**Table 1:** Clinical characteristics of the study population

Characteristic	Cases (n = 390)	Controls (n = 390)	*P*
Age			
Median, years (IQR)	59 (50‐69)	59 (50‐69)	0.893
Gender			
Male	261 (66.9)	261 (66.9)	1.000
Female	129 (33.1)	129 (33.1)	
BMI			
Median, kg/m^2^ (IQR)	24.5 (22.3‐27.7)	24.5 (22.6‐27.4)	0.953
Cigarette smoking status, n (%)			
Never	245 (63.0)	254 (65.1)	0.533
Ever	144 (37.0)	136 (34.9)	
Alcohol consumption, n (%)			
Never	299 (76.9)	220 (56.4)	**<0.001**
Ever	90 (23.1)	170 (43.6)	
Hypertension, n (%)			
No	214 (54.9)	280 (71.8)	**<0.001**
Yes	176 (45.1)	110 (28.2)	
Diabetes, n (%)			
No	314 (80.7)	352 (90.3)	**<0.001**
Yes	75 (19.3)	38 (9.7)	
Stage, n (%)			
I‐II	300 (80.9)		
III‐IV	71 (19.1)		
Grade, n (%)			
I‐II	243 (73.2)		
III‐IV	89 (26.8)		
Follow‐up^a^, n (%)			
Recurrence	20 (7.2)		
Deaths	9 (3.2)		

BMI, body mass index; IQR, interquartile range.

*P* < 0.05 are in boldface.

a With median follow‐up of 19.6 mo.

Of the 25 *GRM* SNPs evaluated, *GRM5* rs7102764 and *GRM7* rs756084 were associated with RCC risk (nominal *P* ≤ 0.049, Table S1). However, only *GRM5* rs7102764 attained significance after adjustment for the false discovery rate (*q* value) at a level of <0.20 (*q* = 0.140, Table 2). In addition, this association persisted after controlling for age, gender, alcohol consumption, and histories of hypertension and diabetes (*P* = 0.006).

**Table 2:** Association between *GRM* SNPs and RCC risk

Gene	SNP	Genotype	Cases, n (%)	Controls, n (%)	OR (95% CI)	*P*	*q*	OR (95% CI)^a^	*P* a
*GRM5*	rs7102764	AA	209 (54.3)	249 (63.8)					
		AT	151 (39.2)	125 (32.1)					
		TT	25 (6.5)	16 (4.1)					
		Trend			1.41 (1.11‐1.79)	0.005	**0.140**	1.42 (1.11‐1.83)	**0.006**
*GRM7*	rs756084	CC	100 (26.0)	119 (30.5)					
		CA	191 (49.6)	197 (50.5)					
		AA	94 (24.4)	74 (19.0)					
		Trend			1.22 (1.00‐1.50)	0.049	0.403		

95% CI, 95% confidence interval; OR, odds ratio; RCC, renal cell carcinoma; SNP, single‐nucleotide polymorphism.

*q* < 0.20 are in boldface.

a ORs were adjusted for age, gender, alcohol consumption, and histories of hypertension and diabetes.

**Figure 1:**
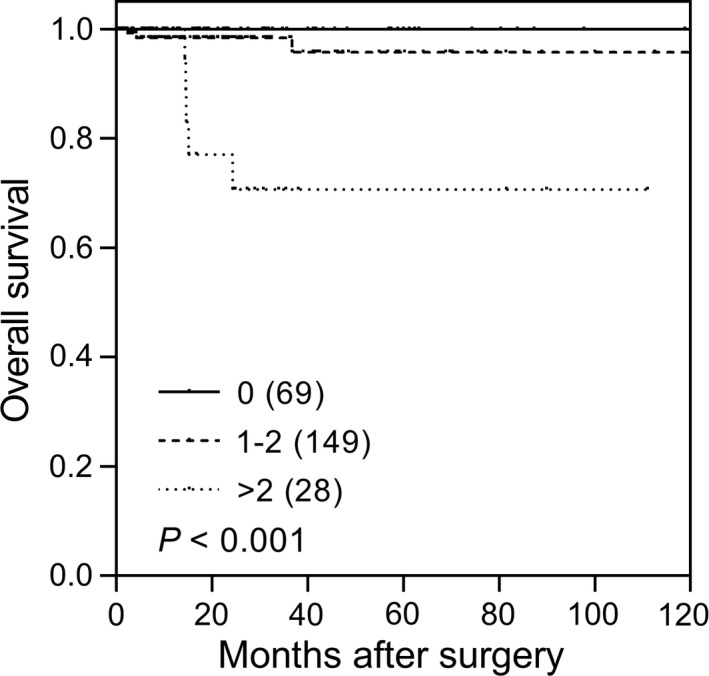
Impact of genetic variants of *GRM3* and *GRM4* on renal cell carcinoma (RCC) survival. Kaplan‐Meier curves of overall survival for RCC patients with 0, 1‐2, or >2 risk alleles (*GRM3* rs701332 C, *GRM4* rs2499707 T, and *GRM4* rs4713742 T). The numbers in parentheses indicate the number of patients

Two SNPs, *GRM3* rs701332 and *GRM4* rs2499707, showed a nominal correlation with RFS, but none of them passed the *q* value threshold (Tables S1 and S2). *GRM3* rs701332, *GRM4* rs2499707, and *GRM4* rs4713742 were associated with OS (nominal *P* ≤ 0.018, Table S1), and all had a *q* value of ≤0.133 (Table 3). A strong gene‐dosage effect on OS was observed when these three SNPs were analysed in combination, and the HRs increased as the number of risk alleles increased (*P* = 0.001, Table 3 and Figure 1).

**Table 3:** Association between *GRM* SNPs and overall survival in patients with RCC

Gene	SNP	Genotype	n of patients	n of events	5‐y survival rate (%)	*P* a	*q*	HR (95% CI)^b^	*P* b
*GRM3*	rs701332	TT	235	5	96.6				
		TC	42	4	81.6				
		Trend				0.015	**0.133**	4.28 (1.15‐16.0)	**0.030**
*GRM4*	rs2499707	CC	192	3	96.7				
		CT	70	4	89.9				
		TT	11	2	70.0				
		Trend				0.001	**0.028**	3.55 (1.46‐8.65)	**0.005**
*GRM4*	rs4713742	CC	117	2	96.1				
		CT	117	2	97.6				
		TT	35	4	82.1				
		Trend				0.018	**0.133**	3.11 (1.17‐8.27)	**0.023**
n of risk alleles									
		0	69	0	100.0				
		1‐2	149	3	95.8				
		>2	28	5	70.6				
		Trend				<0.001		10.4 (2.73‐39.8)	**0.001**

95% CI, 95% confidence interval; HR, hazard ratio; RCC, renal cell carcinoma; SNP, single‐nucleotide polymorphism.

*q* < 0.20 are in boldface.

a *P* values were calculated using the log‐rank test.

b HRs were adjusted for age and gender.

**Figure 2:**
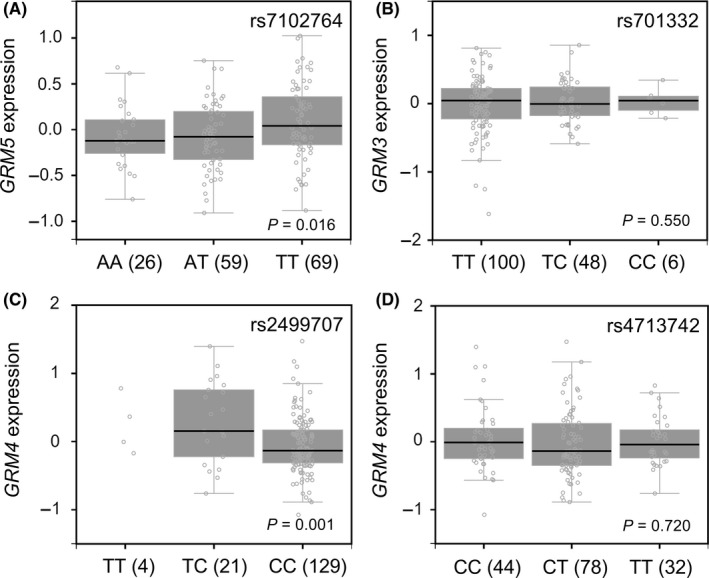
The expression quantitative trait loci analysis of *GRM* SNPs and gene expression levels. Correlations of (A) rs7102764 genotypes with *GRM5* expression, (B) rs701332 genotypes with *GRM3* expression, (C) rs2499707 genotypes with *GRM4* expression, and (D) rs4713742 genotypes with *GRM4* expression. The numbers in parentheses indicate the number of patients

We investigated the functional significance of all genetic variants in linkage disequilibrium with the prognostic SNPs identified in this study using HaploReg (Tables S3‐S6). *GRM5* rs7102764, *GRM3* rs701332, *GRM4* rs2499707, and *GRM4* rs4713742 all coincided with enhancer histone marks in multiple tissues, suggesting that these SNPs might influence the gene expression of *GRM*s. In the eQTL analysis from the GTEx dataset, the risk allele T of rs7102764 showed an increased *GRM5* expression (*P* = 0.016, Figure 2A), and the risk allele T of rs2499707 showed an increased *GRM4* expression (*P* = 0.001, Figure 2C) in most *GRM*s abundantly expressed brain cerebellum tissues.

**Figure 3:**
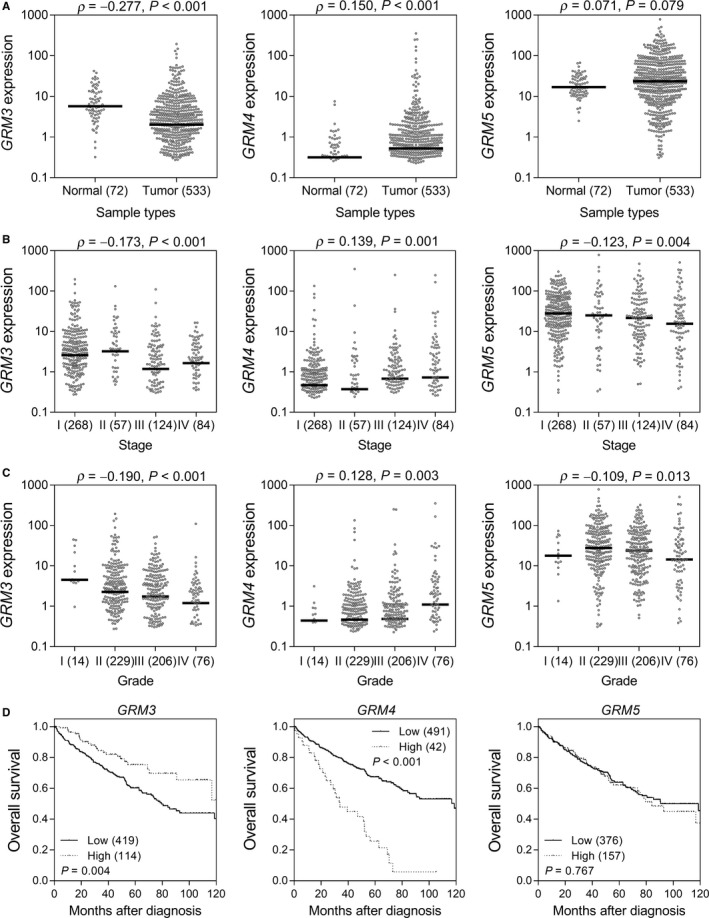
Roles of *GRM3*, *GRM4*, and *GRM5* expression in renal cell carcinoma (RCC) progression. *GRM3*, *GRM4*, and *GRM5* expression levels in (A) normal and tumour tissues, (B) different stages, and (C) different grades of RCC. (D) Kaplan‐Meier curves of overall survival according to *GRM3*, *GRM4*, and *GRM5* expression levels. Patients were dichotomised at the mean gene expression level into the low and high groups. The numbers in parentheses indicate the number of patients

We further determined the clinical relevance of *GRM3*, *GRM4*, and *GRM5* expression in RCC using the TCGA KIRC dataset. *GRM3* gene expression was significantly lower in tumour, late‐stage, and high‐grade tissues (*P* < 0.001, Figure 3A‐C), and low expression of *GRM3 *was associated with poor OS in patients with RCC (*P* = 0.004, Figure 3D). *GRM4* was highly expressed in tumour, late‐stage, and high‐grade tissues (*P* ≤ 0.003), and a high expression of *GRM4 *was associated with poor OS (*P* < 0.001). However, there was only a slight trend towards higher *GRM5* expression levels in cancer tissues than in adjacent normal tissues (*P* = 0.079); *GRM5* expression was not associated with survival.

## 4 DISCUSSION

In the present study, we explored the effects of genetic variants in *GRM*s on the risk and the prognosis of RCC patients. Several significant associations of *GRM3*, *GRM4*, and *GRM5* with RCC susceptibility and survival were identified. These findings highlight the importance of GRMs in RCC and might have the potential to guide the selection of patients at a high risk of poor prognosis.

Our results indicated that *GRM5* rs7102764, an intronic variant, was associated with RCC risk. Functional prediction implicated this variant as an eQTL regulating the expression of *GRM5*, potentially through the modulation of the enhancer activities and transcription‐factor binding affinities. A tendency of *GRM5* gene upregulation was observed in tumour tissues, suggesting that this gene may play a role in RCC carcinogenesis. Studies have shown that GRM5 is upregulated in lung and glial cancers,^20^, ^21^ and inactivation of GRM5 suppresses liver and bone cancer cell proliferation by blocking mitogen‐activated protein kinase pathways.^22^, ^23^ The intronic variants rs701332 located in *GRM3*, and rs2499707 and rs4713742 located in *GRM4*, were associated with the survival of RCC patients. According to the GTEx dataset, rs2499707 is an eQTL that affects the expression of *GRM4*. In addition, downregulation of *GRM3* was observed in tumour tissues and correlated with a shorter survival of RCC patients, whereas *GRM4* overexpression was found in tumours and correlated with poor survival. Consistent with our findings, it has been shown that GRM3 mediates B‐cell‐related tumour cell apoptosis via forkhead box O1, and GRM3 deficiency promotes tumour progression.^24^ Mounting evidence has also revealed that GRM4 overexpression is correlated with poor prognosis in various cancers such as osteosarcoma,^25^ colorectal cancer,^9^ and glioma.^26^ Further mechanistic studies have demonstrated that transcriptional misregulation in cancers and peroxisome proliferator‐activated receptor signalling pathways might participate in GRM4‐mediated osteosarcoma progression.^27^ Taken together, the genetic variants identified in this study might affect the gene expression of GRMs, which in turn influence the progression of RCC through modulating tumour cell apoptosis by GRM3, as well as misregulating peroxisome proliferator‐activated receptor signalling pathway by GRM4, and mitogen‐activated protein kinase pathway by GRM5. However, the mechanisms underlying the effects of GRMs in RCC remain undetermined and warrant further investigations.

Several limitations should be noted with regard to interpreting the results of our study. First, the sample size was relatively small, the follow‐up time was limited, and the frequencies of recurrent/death events and some homozygous variants were low in subgroups, which could limit the accuracy and reliability of our results. Second, our findings may not be generalized to other ethnicities as the study population was mainly Taiwanese; however, similar genetic backgrounds can minimise the potential confounding of population heterogeneity. Third, the SNPs genotyped in this study were haplotype‐tagging SNPs to capture most of the genomic diversity; however, the linked causal SNPs and exact molecular mechanisms need to be further identified. Fourth, due to the low number (N = 39) of kidney tissue samples in the GTEx dataset, our eQTL analyses were limited to the brain cerebellum, in which most *GRM*s are abundantly expressed, but not the target tissues. Finally, although false discovery rates were reported to account for multiple hypothesis testing, we still cannot rule out the possibility of false‐positive findings. However, functional studies support the clinical significance of GRMs in RCC. To our knowledge, this study is the first attempt to discover the effect of GRMs on RCC development and progression. The results are intriguing and worth replicating in further independent studies with larger sample size, as well as performing functional experiments to confirm our findings.

In conclusion, we identified multiple novel associations of genetic variants in *GRM3*, *GRM4*, and *GRM5* with the risk and the survival of RCC. Furthermore, these relationships were supported by gene expression profiles obtained using bioinformatics analysis. Specifically, the expression of *GRM4* and *GRM5* showed an increasing trend in RCC tissues, compared to that in normal tissue, whereas *GRM3* was downregulated in RCC. Consistently, the increased expression of *GRM4* and decreased expression of *GRM3* were associated with poorer survival in patients with RCC. Collectively, these results provide evidence in support of the hypothesis that GRMs modulate the development and progression of RCC.

## CONFLICT OF INTEREST

The authors have no conflicts of interest to declare.
