# Supplementary material for: Clinical significance of glutamate metabotropic receptors in renal cell carcinoma risk and survival
Source: Cancer Med. 2018 Nov 28;7(12):6104–11. doi: 10.1002/cam4.1901 (PMC6308098; doi:10.1002/cam4.1901)
Supplement: Supplementary file 1 [file CAM4-7-6104-s001.doc]

**Table S1.** Genotyped SNPs and the *P* values of their association with RCC risk, RFS, and OS

| Gene | SNP ID | Chromosome | Position | Risk^a^ | RFS^b^ | OS^b^ |
| --- | --- | --- | --- | --- | --- | --- |
| *GRM1* | rs7761033 | 6 | 146397799 | 0.361 | 0.881 | 0.175 |
| *GRM1* | rs17075819 | 6 | 146618510 | 0.902 | 0.270 | 0.87 |
| *GRM2* | rs4067 | 3 | 51713296 | 0.843 | 0.161 | 0.264 |
| ***GRM3*** | **rs701332** | 7 | 86115570 | 0.694 | **0.048** | **0.015** |
| *GRM3* | rs6943659 | 7 | 86233862 | 0.689 | 0.571 | 0.050 |
| *GRM3* | rs10266758 | 7 | 86280430 | 0.973 | 0.675 | 0.655 |
| *GRM4* | rs2499674 | 6 | 34113954 | 0.961 | 0.563 | 0.701 |
| *GRM4* | rs1873254 | 6 | 34134467 | 0.586 | 0.984 | 0.770 |
| *GRM4* | rs2260612 | 6 | 34150536 | 0.310 | 0.539 | 0.734 |
| ***GRM4*** | **rs2499707** | 6 | 34161644 | 0.878 | **0.039** | **0.001** |
| ***GRM4*** | **rs4713742** | 6 | 34197572 | 0.262 | 0.424 | **0.018** |
| *GRM5* | rs10741500 | 11 | 88075857 | 0.785 | 0.625 | 0.232 |
| ***GRM5*** | **rs7102764** | 11 | 88150908 | **0.005** | 0.623 | 0.911 |
| *GRM6* | rs762724 | 5 | 178349488 | 0.678 | 0.128 | 0.085 |
| *GRM6* | rs7733067 | 5 | 178358320 | 0.786 | 0.076 | 0.640 |
| *GRM7* | rs1553149 | 3 | 7170734 | 0.621 | 0.356 | 0.560 |
| *GRM7* | rs1396405 | 3 | 7340432 | 0.056 | 0.687 | 0.530 |
| *GRM7* | rs17717959 | 3 | 7456854 | 0.653 | 0.277 | 0.504 |
| ***GRM7*** | **rs756084** | 3 | 7536149 | **0.049** | 0.259 | 0.124 |
| *GRM7* | rs9311994 | 3 | 7646655 | 0.330 | 0.824 | 0.537 |
| *GRM8* | rs4236623 | 7 | 126084314 | 0.794 | 0.108 | 0.703 |
| *GRM8* | rs1419465 | 7 | 126346234 | 0.464 | 0.624 | 0.099 |
| *GRM8* | rs3779538 | 7 | 126486324 | 0.637 | 0.308 | 0.930 |
| *GRM8* | rs17862281 | 7 | 126494939 | 0.937 | 0.914 | 0.506 |
| *GRM8* | rs6942923 | 7 | 126506071 | 0.286 | 0.695 | 0.668 |

Abbreviations: SNP, single nucleotide polymorphism; RCC, renal cell carcinoma; RFS, recurrence-free survival; OS, overall survival.

^a^*P* values were calculated using the logistic regression.

^b^*P* values were calculated using the log-rank test.

*P* < 0.05 is in boldface.

**Table S2.** Association between *GRM* SNPs and disease recurrence in patients with RCC

| Gene | SNP | Genotype | n of patients | n of events | 5-year survival rate (%) | *P*^a^ | *q* |
| --- | --- | --- | --- | --- | --- | --- | --- |
| *GRM3* | rs701332 | TT | 236 | 13 | 91.1 |  |  |
|  |  | TC | 41 | 7 | 68.4 |  |  |
|  |  | Trend |  |  |  | 0.048 | 0.672 |
| *GRM4* | rs2499707 | CC | 191 | 9 | 88.5 |  |  |
|  |  | CT | 70 | 9 | 81.1 |  |  |
|  |  | TT | 12 | 2 | 66.7 |  |  |
|  |  | Trend |  |  |  | 0.039 | 0.672 |

Abbreviations: SNP, single nucleotide polymorphism; RCC, renal cell carcinoma.

^a^*P* values were calculated using the log-rank test.

**Table S3.** Regulatory annotation of variants linked with *GRM5* rs7102764

| Chromosome | Position | LD (r^2^) | SNP ID | Reference allele | Alternate allele | ASN frequency | Variant type | Promoter histone marks | Enhancer histone marks | DNAse | Motifs changed |
| --- | --- | --- | --- | --- | --- | --- | --- | --- | --- | --- | --- |
| 11 | 88751974 | 0.93 | rs12276694 | G | A | 0.25 | Intronic |  |  |  | Foxp1, Mef2, TATA |
| 11 | 88753467 | 0.92 | rs34096746 | G | A | 0.23 | Intronic |  |  |  | Pax-4 |
| 11 | 88756258 | 0.98 | rs11604767 | T | C | 0.24 | Intronic |  |  |  | Hoxa10, Irx, Pou2f2 |
| 11 | 88757237 | 0.98 | rs10831366 | C | T | 0.24 | Intronic |  |  |  | EBF, STAT |
| 11 | 88757676 | 0.98 | rs2126741 | A | G | 0.24 | Intronic |  |  |  | AP-2rep, BCL, MOVO-B, Znf143 |
| 11 | 88758547 | 0.9 | rs1603895 | A | G | 0.23 | Intronic |  |  |  | BCL, Foxa, Foxd3, Foxo, Foxp1, HDAC2, HMG-IY, Irf, RXRA, Zfp105, p300 |
| 11 | 88760362 | 0.94 | rs34066725 | A | G | 0.23 | Intronic |  |  |  | Evi-1, Foxp1, Mef2, RXR::LXR, STAT, Sox, p300 |
| 11 | 88761708 | 0.96 | rs2201930 | A | C | 0.24 | Intronic |  |  |  | Foxa, Hand1, Mef2, Pou3f2, STAT |
| 11 | 88761785 | 0.94 | rs2201928 | A | G | 0.23 | Intronic |  |  |  | ATF3, SREBP, p300 |
| 11 | 88763555 | 0.83 | rs34477757 | TG | T | 0.21 | Intronic |  |  |  | FAC1, Pou2f2 |
| 11 | 88764594 | 0.98 | rs35531644 | CT | C | 0.24 | Intronic |  |  |  | Pbx-1, Smad3, Sox |
| 11 | 88764597 | 0.96 | rs66672629 | AG | A | 0.24 | Intronic |  |  |  | Evi-1, Foxd3, Foxo, Foxp1, HDAC2, Pbx-1, Sox |
| 11 | 88766154 | 0.82 | rs12785875 | G | A | 0.21 | Intronic |  |  |  |  |
| 11 | 88769356 | 0.94 | rs34729934 | A | G | 0.23 | Intronic |  |  |  | Gm397, Pou3f3 |
| 11 | 88771886 | 0.94 | rs12222022 | C | T | 0.23 | Intronic |  |  |  | Gfi1, Mxi1, Pou5f1, RBP-Jkappa, SREBP |
| 11 | 88775023 | 1 | rs144645528 | G | A | 0.24 | Intronic |  |  |  | Pax-4, Pax-6, p300 |
| 11 | 88776551 | 1 | rs7924618 | T | A | 0.24 | Intronic |  |  |  | Hoxa3 |
| 11 | 88777105 | 0.98 | rs2387410 | T | C | 0.24 | Intronic |  |  |  | RBP-Jkappa |
| 11 | 88777302 | 1 | rs1909399 | C | T | 0.24 | Intronic |  |  |  | Nrf1, SRF |
| 11 | 88777458 | 0.96 | rs56273627 | T | C | 0.23 | Intronic |  |  |  | Nrf1 |
| 11 | 88778092 | 1 | **rs7102764** | A | T | 0.24 | Intronic |  | SKIN |  | ERalpha-a, Esr2, RXRA |
| 11 | 88779534 | 1 | rs12280431 | A | T | 0.24 | Intronic |  | FAT, STRM, BLD, SKIN, BONE | SKIN, SKIN, SKIN, LNG | Pou2f2, Pou5f1 |
| 11 | 88780506 | 0.96 | rs11021304 | C | T | 0.23 | Intronic |  | FAT, BONE |  | AP-3, Sox, YY1 |
| 11 | 88781854 | 0.98 | rs7119749 | A | G | 0.24 | Intronic |  | SKIN | ESC, ESC, IPSC, IPSC, SKIN, SKIN | SPIB, STAT |
| 11 | 88786761 | 0.94 | rs17182184 | G | T | 0.23 | Intronic |  |  |  | Cdx, Foxa, Foxc1, Foxd1, Foxp1, HDAC2, HNF1, Hoxa10, Pou2f2 |
| 11 | 88787639 | 0.98 | rs3913309 | G | A | 0.24 | Intronic |  |  |  | Gfi1 |
| 11 | 88789575 | 0.8 | rs3913310 | C | T | 0.21 | Intronic |  |  |  | Hltf, Hoxa9, Hoxb9, Hoxd10, Obox6 |
| 11 | 88790291 | 0.96 | rs10831438 | A | G | 0.24 | Intronic |  |  |  | GR |
| 11 | 88793573 | 0.98 | rs10765773 | G | C | 0.24 | Intronic |  |  |  | Arid3a, CHX10, Cart1, Cdx2, Hoxa10, Hoxa3, Hoxa5, Hoxa7, Hoxa9, Hoxb13, Hoxb6, Hoxb8, Hoxc9, Hoxd10, Lhx3, Nkx6-1, Nobox, Pax7, Pou3f2, Pou4f3, TCF4 |
| 11 | 88798609 | 0.98 | rs10501686 | G | A | 0.76 | Intronic |  |  |  | Arid5a, Hoxc10 |
| 11 | 88799011 | 0.94 | rs10501687 | T | C | 0.23 | Intronic |  |  |  | Gfi1, Gfi1b |
| 11 | 88799115 | 0.92 | rs4753199 | C | T | 0.75 | Intronic |  | BRN | OVRY | HNF4, Pax-6 |
| 11 | 88800692 | 0.98 | rs3907594 | C | G | 0.76 | Intronic |  | BLD, BRN, SKIN, LIV, GI | GI | GR, HNF1, TATA |
| 11 | 88801547 | 0.98 | rs1813119 | T | C | 0.76 | Intronic |  | ESC, BLD, LIV, GI |  | Foxc1, Foxd1, Foxf1, Foxj1, Foxk1, Foxo, Foxq1, HNF1, Irx, Nkx2, OTX, Pbx-1, Pou1f1 |
| 11 | 88804453 | 0.94 | rs35013840 | G | A | 0.23 | Intronic |  |  |  | GR, Pax-4, Pou2f2, RREB-1 |
| 11 | 88807016 | 0.98 | rs2169660 | G | A | 0.76 | Intronic |  | BLD |  | DMRT2, Irf, PLZF, STAT, Sox |
| 11 | 88810547 | 0.94 | rs12787863 | C | T | 0.23 | Intronic |  |  |  | Hoxa7, Mrg1::Hoxa9 |
| 11 | 88810588 | 0.98 | rs6483475 | A | C | 0.76 | Intronic |  |  |  | Foxa, Foxl1 |
| 11 | 88811846 | 0.94 | rs12795125 | C | T | 0.23 | Intronic |  | LIV | IPSC | Hic1 |
| 11 | 88813248 | 0.98 | rs1027533 | T | C | 0.76 | Intronic |  |  | ESC, IPSC, BLD | Foxi1, Foxp1, Foxq1, Irx, Pou1f1 |
| 11 | 88815680 | 0.98 | rs3956251 | C | G | 0.76 | Intronic |  |  | ESC, BLD, BLD | Ets, GATA, RXRA, SP1, TBX5 |
| 11 | 88818713 | 0.96 | rs10765794 | C | G | 0.76 | Intronic |  |  |  | CEBPB, GR, Nanog, Pou5f1, Sox |
| 11 | 88819213 | 0.96 | rs7109255 | A | G | 0.76 | Intronic |  | LIV | ADRL | ERalpha-a, HNF4, NR4A, Nr2f2, RAR, RXRA |
| 11 | 88819943 | 0.98 | rs6483483 | T | C | 0.76 | Intronic |  | LIV |  | Nanog, Pou5f1 |
| 11 | 88821002 | 0.98 | rs7479952 | C | T | 0.76 | Intronic |  |  |  | CEBPG, Maf, TATA |
| 11 | 88821586 | 0.98 | rs6416019 | T | G | 0.76 | Intronic |  |  |  | Mrg, Myb |
| 11 | 88824028 | 0.98 | rs10765797 | T | G | 0.76 | Intronic |  |  |  | Foxa, HNF1, Pou1f1, Pou2f2 |
| 11 | 88824823 | 0.98 | rs10831496 | A | G | 0.76 | Intronic |  |  |  | RREB-1, SRF |
| 11 | 88825308 | 0.94 | rs2047512 | T | C | 0.23 | Intronic |  |  |  | Foxd3, Foxp1, Sox |
| 11 | 88826381 | 0.98 | rs10741530 | A | C | 0.76 | Intronic |  |  |  | CDP, Dbx1, Hdx, Homez, TATA |
| 11 | 88826760 | 0.98 | rs11021475 | C | G | 0.76 | Intronic |  | LIV |  | Evi-1 |
| 11 | 88827816 | 0.98 | rs1499185 | C | G | 0.76 | Intronic |  | LIV |  |  |
| 11 | 88828124 | 0.98 | rs1499184 | G | C | 0.76 | Intronic |  |  | THYM | GR |
| 11 | 88829179 | 0.82 | rs200619793 | T | TG | 0.74 | Intronic |  |  |  | Hic1, Pax-5, Zec, p53 |
| 11 | 88829742 | 0.94 | rs2648640 | A | G | 0.77 | Intronic |  |  |  | DMRT5, FAC1, Foxp1, Sox |
| 11 | 88829863 | 0.8 | rs17184781 | G | A | 0.21 | Intronic |  |  |  | Maf, Pou2f2 |
| 11 | 88831365 | 0.98 | rs480015 | A | C | 0.76 | Intronic |  |  |  | HNF1, Nr2e3, Znf143 |
| 11 | 88834601 | 0.96 | rs202011961 | G | GCA | 0.76 | Intronic |  |  |  | Arnt, BHLHE40, DMRT1, Mxi1, Myc, ZEB1 |
| 11 | 88834604 | 0.96 | rs33977147 | C | CAT | 0.76 | Intronic |  |  |  | Arnt, BHLHE40, DMRT1, Mxi1, Myc, Myf, RP58 |
| 11 | 88835211 | 0.95 | rs492312 | G | A | 0.76 | Intronic |  | BLD |  | COMP1, STAT |
| 11 | 88835513 | 0.94 | rs578697 | C | T | 0.76 | Intronic |  | BLD |  | BATF, E2F, Gfi1, Irf, NF-Y, Pbx3 |
| 11 | 88838336 | 0.96 | rs586927 | T | C | 0.76 | Intronic |  |  | ESDR, ESDR, LNG, BRST, SKIN, SKIN, BRST, BRN, SKIN, LNG | AP-1, AP-2, BAF155, BATF, BCL, Bach1, Bach2, E2A, GR, HMGN3, Irf, KAP1, Mef2, Myc, NF-E2, Nrf-2, PRDM1, RXRA, STAT, Smad3, TCF4, ZEB1, p300 |
| 11 | 88843838 | 0.91 | rs11021570 | A | G | 0.23 | Intronic |  | BLD, THYM | BLD, BLD, BLD, THYM | Foxp3 |
| 11 | 88847303 | 0.85 | rs7927169 | T | C | 0.23 | Intronic |  | BLD |  | Cart1, Dbx1, Foxd3, Foxp1, Hoxd8, Pax-4, Sox, Zfp105 |
| 11 | 88849440 | 0.85 | rs59185944 | G | A | 0.23 | Intronic |  |  | SKIN, SKIN, BRST, SKIN, LNG | Cdx2, HNF1, Hoxa10, Hoxc10, Hoxd10 |
| 11 | 88850998 | 0.87 | rs7939942 | T | C | 0.23 | Intronic |  |  |  | Sox |

**Table S4.** Regulatory annotation of variants linked with *GRM3* rs701332

| Chromosome | Position | LD (r^2^) | SNP ID | Reference allele | Alternate allele | ASN frequency | Variant type | Promoter histone marks | Enhancer histone marks | DNAse | Motifs changed |
| --- | --- | --- | --- | --- | --- | --- | --- | --- | --- | --- | --- |
| 7 | 86648318 | 1 | **rs701332** | T | C | 0.06 | Intronic | BRN | ESC, ESDR, IPSC, BRN | ESDR | DMRT2 |
| 7 | 86653810 | 0.97 | rs802429 | A | G | 0.06 | Intronic | BRN | ESDR, ESC, IPSC, BRN |  | Arid5a, Dbx1, Dbx2, GATA, Hoxd10, Hoxd8, Ncx, Pou1f1, Pou2f2, Pou3f2, Pou3f3, Pou4f3, TAL1, TATA |
| 7 | 86655117 | 1 | rs802432 | A | G | 0.06 | Intronic |  | BRN |  | Hoxa7 |
| 7 | 86666433 | 0.93 | rs10257201 | T | C | 0.05 | Intronic |  |  |  | Pou1f1 |
| 7 | 86676383 | 0.93 | rs7806940 | A | G | 0.05 | Intronic |  | BRN |  | CIZ, Foxp1, HMG-IY, HNF1, IRC900814, Ncx, Sox, Zfp105 |
| 7 | 86680384 | 0.9 | rs34952279 | CA | C | 0.05 | Intronic |  |  |  | Foxa, GR, Mef2, YY1, Zfp128 |
| 7 | 86685104 | 0.93 | rs13242038 | C | T | 0.05 | Intronic |  |  | OVRY | Foxa |
| 7 | 86692495 | 0.93 | rs10227921 | C | A | 0.05 | Intronic |  |  |  | ATF3, HEY1, TATA |
| 7 | 86693703 | 0.93 | rs11762187 | G | A | 0.05 | Intronic |  |  |  | GATA |
| 7 | 86695952 | 0.93 | rs12704282 | T | C | 0.05 | Intronic |  |  |  | Foxd1 |
| 7 | 86700414 | 0.93 | rs1527762 | C | A,T | 0.05 | Intronic |  | BRN |  |  |
| 7 | 86706496 | 0.93 | rs2299218 | C | T | 0.05 | Intronic |  | ESDR, BRN | IPSC, ADRL, BRN | HNF1 |
| 7 | 86706934 | 0.93 | rs6950735 | A | C | 0.05 | Intronic |  | ESDR, BRN | BRN, BRN |  |

**Table S5.** Regulatory annotation of variants linked with *GRM4* rs2499707

| Chromosome | Position | LD (r^2^) | SNP ID | Reference allele | Alternate allele | ASN frequency | Variant type | Promoter histone marks | Enhancer histone marks | DNAse | Motifs changed |
| --- | --- | --- | --- | --- | --- | --- | --- | --- | --- | --- | --- |
| 6 | 34083748 | 1 | rs2499705 | G | C | 0.83 | Intronic |  | ESC, ESDR, IPSC, BRN, MUS |  | TCF4, TFII-I |
| 6 | 34084435 | 0.91 | rs2499706 | G | A | 0.81 | Intronic |  | ESC, ESDR, IPSC, BRN |  | AP-2, CIZ, MZF1::1-4, PTF1-beta, Pax-4 |
| 6 | 34085889 | 1 | **rs2499707** | T | C | 0.83 | Intronic |  | IPSC, SPLN |  |  |
| 6 | 34088949 | 1 | rs2499711 | T | G | 0.83 | Intronic |  | SKIN |  | CTCF, E2A, RXRA, Rad21, SMC3, TCF12 |
| 6 | 34093476 | 0.9 | rs2451351 | A | G | 0.84 | Intronic |  | ESC, PLCNT, CRVX |  | CTCF, Maf, NRSF, RXRA, Rad21, SMC3 |
| 6 | 34095574 | 0.9 | rs2499713 | T | C | 0.84 | Intronic |  | ESC |  | BCL, BDP1, BHLHE40, HDAC2, NRSF, Sin3Ak-20 |
| 6 | 34098847 | 0.88 | rs2451348 | T | C | 0.84 | Intronic |  | ESC, ESDR, IPSC |  | Egr-1, PPAR, Pou2f2, SRF, WT1, Zfp281 |

**Table S6.** Regulatory annotation of variants linked with *GRM4* rs4713742

| Chromosome | Position | LD (r^2^) | SNP ID | Reference allele | Alternate allele | ASN frequency | Variant type | Promoter histone marks | Enhancer histone marks | DNAse | Motifs changed |
| --- | --- | --- | --- | --- | --- | --- | --- | --- | --- | --- | --- |
| 6 | 34105630 | 0.88 | rs11753413 | C | T | 0.44 | Intronic |  | IPSC, GI |  |  |
| 6 | 34105997 | 0.96 | rs12525536 | C | T | 0.42 | Intronic |  | GI | GI | SETDB1, SP1, TATA |
| 6 | 34106002 | 0.88 | rs12528906 | A | G | 0.44 | Intronic |  | GI | GI | SP1, SP2, Spz1 |
| 6 | 34106477 | 0.8 | rs11447641 | G | GT | 0.46 | Intronic |  |  |  | Evi-1, PEBP, PTF1-beta |
| 6 | 34106686 | 0.86 | rs1109654 | A | G | 0.45 | Intronic |  |  |  | CTCF, Hbp1 |
| 6 | 34107777 | 0.8 | rs1075482 | A | G | 0.47 | Intronic |  |  |  | CTCF, WT1, ZBTB33 |
| 6 | 34114558 | 0.96 | rs9368794 | A | G | 0.42 | Intronic |  | IPSC, BRN, SPLN |  | ELF1, Ets, HMG-IY, LBP-9, PPAR, SETDB1, ZBTB7A |
| 6 | 34115167 | 0.95 | rs12205473 | G | A | 0.43 | Intronic | SKIN | IPSC, SKIN, BRN, SPLN |  | KAP1 |
| 6 | 34115682 | 0.81 | rs2499722 | C | T | 0.46 | Intronic |  | BRN, MUS | ESDR, ESC, IPSC, BRN, BRN, HRT, GI | Ets |
| 6 | 34117036 | 0.97 | rs4713739 | C | G | 0.42 | Intronic |  |  |  | EBF, PLAG1 |
| 6 | 34117085 | 0.82 | rs4713740 | A | C | 0.47 | Intronic |  |  |  | Pou5f1 |
| 6 | 34117258 | 0.98 | rs4713741 | G | T | 0.42 | Intronic |  |  |  | BCL, Mef2, NF-kappaB, Otx2, Spz1 |
| 6 | 34119035 | 0.91 | rs2499725 | A | C | 0.44 | Intronic |  |  |  | Brachyury, Eomes, Mrg, SIX5, TBX5, Tgif1 |
| 6 | 34119859 | 0.91 | rs2499726 | A | G | 0.44 | Intronic |  |  |  | Egr-1, MOVO-B, Pou1f1, SRF, TATA, YY1 |
| 6 | 34120879 | 0.92 | rs923498 | C | T | 0.44 | Intronic |  | ESC, IPSC, GI |  | AP-1, AP-2, BAF155, BATF, BCL, Bach1, Bach2, GATA, GR, HMGN3, KAP1, Mef2, Myc, PRDM1, RXRA, SETDB1, STAT, TCF4, Znf143, p300 |
| 6 | 34121509 | 0.92 | rs2451339 | C | G | 0.44 | Intronic |  | ESC, IPSC, MUS, PLCNT, GI | ESC, ESDR, ESC, IPSC, IPSC, ADRL | HEY1, LBP-1, LBP-9, PLAG1 |
| 6 | 34121817 | 1 | **rs4713742** | C | T | 0.42 | Intronic |  | ESC, IPSC, SKIN, PLCNT, GI |  | BRCA1, Ets, Myf, PU.1, Pax-5, TBX5 |
| 6 | 34121842 | 1 | rs4713743 | G | A | 0.42 | Intronic |  | ESC, IPSC, GI |  |  |
| 6 | 34122707 | 0.92 | rs2499728 | C | T | 0.41 | Intronic |  | IPSC, GI |  | AP-2, CACD, EBF, HEY1, Rad21 |
| 6 | 34122784 | 0.96 | rs2451337 | G | C | 0.42 | Intronic |  | IPSC, GI |  | EWSR1-FLI1, MOVO-B, MZF1::1-4, Zfp740 |
| 6 | 34123388 | 0.98 | rs9394188 | C | T | 0.42 | Intronic |  | IPSC |  | CACD, SP2, ZBTB7A |
| 6 | 34124130 | 0.98 | rs9394189 | C | A | 0.42 | Intronic |  | IPSC |  | Tgif1 |
| 6 | 34129774 | 0.94 | rs2499729 | C | T | 0.42 | Intronic |  | ESC, ESDR, IPSC | ESC, ESDR, ESC, IPSC, IPSC |  |
| 6 | 34134284 | 0.94 | rs2499730 | G | T | 0.42 | Intronic |  | ESC, IPSC |  | ERalpha-a, Ets, Mtf1, Pax-5, Pax-8, p300 |
| 6 | 34141250 | 0.88 | rs2499732 | T | C | 0.58 | Intronic |  | ESC, IPSC, BRST, MUS |  | AP-4, Ascl2, E2A, EBF, HEN1, LBP-1, Lmo2-complex, Myf, NRSF, Sin3Ak-20, TCF12, p300 |
| 6 | 34141627 | 0.85 | rs6928019 | A | T | 0.42 | Intronic |  | ESC, IPSC, BRST, MUS |  | NF-AT |
